# Achieving “Non-Foaming” Rhamnolipid Production and Productivity Rebounds of *Pseudomonas aeruginosa* under Weakly Acidic Fermentation

**DOI:** 10.3390/microorganisms10061091

**Published:** 2022-05-25

**Authors:** Zhijin Gong, Qiuhong He, Jinfeng Liu, Jing Zhou, Chengchuan Che, Meiru Si, Ge Yang

**Affiliations:** College of Life Sciences, Qufu Normal University, Qufu 273165, China; gongzhijin@126.com (Z.G.); jinfeng101@126.com (J.L.); jingzhou-2004@163.com (J.Z.); chechengchuan@qfnu.edu.cn (C.C.); smr1016@126.com (M.S.); yangge@qfnu.edu.cn (G.Y.)

**Keywords:** rhamnolipids, *Pseudomonas aeruginosa*, weakly acidic fermentation, “non-foaming” fermentation, productivity rebounds, mechanisms of “non-foaming” fermentation

## Abstract

The rhamnolipid production of *Pseudomonas aeruginosa* has been impeded by its severe foaming; overcoming the bottleneck of foaming has become the most urgent requirement for rhamnolipid production in recent decades. In this study, we performed rhamnolipid fermentation under weakly acidic conditions to address this bottleneck. The results showed that the foaming behavior of rhamnolipid fermentation broths was pH-dependent with the foaming ability decreasing from 162.8% to 28.6% from pH 8 to 4. The “non-foaming” rhamnolipid fermentation can be realized at pH 5.5, but the biosynthesis of rhamnolipids was significantly inhibited. Further, rhamnolipid yield rebounded from 8.1 g/L to 15.4 g/L after ultraviolet and ethyl methanesulfonate compound mutagenesis. The mechanism study showed that the species changes of rhamnolipid homologs did not affect the foaming behavior of the fermentation but had a slight effect on the bioactivity of rhamnolipids. At pH 8.0 to 5.0, increased surface tension, decreased viscosity and zeta potential, and aggregation of rhamnolipid molecules contributed to the “non-foaming” rhamnolipid fermentation. This study provides a promising avenue for the “non-foaming” rhamnolipid fermentation and elucidates the mechanisms involved, facilitating the understanding of pH-associated foaming behavior and developing a more efficient strategy for achieving rhamnolipid production.

## 1. Introduction

Rhamnolipids are a class of glycolipid-type biosurfactants produced primarily by *Pseudomonas aeruginosa* [1]. As the most studied biosurfactants, rhamnolipids not only have the properties of solubilization, emulsification, wetting, foaming, dispersion and reduction of surface tension common to surfactants, but also have the advantages of non-toxicity, biodegradability, ecological safety and high surface activity compared with other surfactants produced by chemical synthesis or petroleum refining methods [2,3,4,5,6], and they have received green certifications in the fields of pesticides and food. The U.S. Environmental Protection Agency (EPA) has passed the application and approval of rhamnolipid biosurfactants in food and pesticide chemicals (document citation: 68 FR 25026 (https://www.federalregister.gov/documents/2003/05/09/03-11478/rhamnolipid-biosurfactant-notice-of-filing-a-pesticide-petition-to-establish-a-tolerance-for-a, accessed on 1 May 2022) and 69 FR 16796 (https://www.federalregister.gov/documents/2004/03/31/04-6933/rhamnolipid-biosurfactant-exemption-from-the-requirement-of-a-tolerance, accessed on 1 May 2022)). However, despite their potential, the industrial application of rhamnolipids is seriously restricted because the cost of rhamnolipid production is obviously higher than that of synthetic surfactants [6]. One of the main reasons for the high cost of rhamnolipid production is due to the heavy foaming in rhamnolipid fermentation, which reduces the working volume of the fermenter [3,7], loses cells and media [7] and increases the risk of contamination [7], leading to a significant decrease in fermentation efficiency.

A considerable number of fermentation strategies have been employed to address foaming problems, including the use of defoamers [3], foam fractionation fermentation [8,9,10,11], foam adsorption fermentation [12,13], fermentation–defoaming tandem systems [14], anaerobic fermentation [8] and solid fermentation [9,10,11]. However, traditional chemical defoaming methods such as the use of defoamers have been proved to be ineffective in suppressing severe foaming in rhamnolipid fermentation unless chemical defoamers are used excessively and the working volume of the fermenter is severely sacrificed. Foam fractionation fermentation, foam adsorption fermentation and fermentation–defoaming tandem systems are all ex situ and require external equipment for foam storage or defoaming, causing some other problems such as increased equipment costs, loss of biomass and media and increased contamination risks, which cannot be ignored in large-scale industrial production [1]. Anaerobic and solid fermentations are usually not feasible in the industrial production of rhamnolipids because of their remarkable low productivity [1]. Therefore, submerged liquid fermentation without external equipment (in situ) is still the sole promising method for the large-scale production of rhamnolipids [12]. Determining how to control the foaming of submerged liquid rhamnolipid fermentation has become an urgent requirement for the large-scale production and application of rhamnolipids [13]. In this respect, an in situ rhamnolipid fermentation was recently performed by controlling the pH of fermentation under weakly acidic conditions, achieving low-foam fermentation of rhamnolipids and maximizing cell growth and rhamnolipid productivity, which provides a promising strategy for the production of rhamnolipids by conventional submerged liquid fermentation without the external cost of equipment [14]. In this in situ rhamnolipid fermentation, the cell growth was significantly limited with a maximum cell concentration of only 1.8 g/L at pH 5.1, but when the fermentation was controlled at pH 5.5, the cell growth was decreased by only 20% and rhamnolipid production reached about 15 g/L at low foaming, which is promising for rhamnolipid production. Nevertheless, other studies using weakly acidic fermentation of rhamnolipids were generally unsatisfactory, because although the foaming ability of rhamnolipid fermentation broth was conspicuously eliminated, cell growth and rhamnolipid productivity were also significantly inhibited. Typically, when the pH decreased from 7.0 to 6.5, the cell dry-weight concentration and the rhamnolipid production decreased from approximately 1.8 g/L to 0.36 g/L and 1.2 g/L to 0.2 g/L, respectively [15]. In addition, there are still few studies on rhamnolipid fermentation using the weakly acidic conditions, and even fewer basic studies on systematic evaluation of foaming properties and the low-foaming mechanism of rhamnolipid fermentation under weakly acidic conditions, restricting the better understanding and utilization of weakly acidic conditions to control the foaming in rhamnolipid fermentation.

Therefore, the objectives of this study are to realize low- or non-foaming fermentation and productivity rebounds of rhamnolipids by using weakly acidic fermentation to address the constraints of severe foaming in aerobic rhamnolipid production. In addition, the mechanisms of low- or non-foaming fermentation are also systematically explored by characterizing the effects of weakly acidic conditions on viscosity, surface tension, zeta potential, foaming properties and aggregation behavior of rhamnolipids. Although this research is a conventional bioprocess engineering study, its results are of great value as a baseline for the meaningful development of advanced and unique rhamnolipid production processes in the future.

## 2. Materials and Methods 

### 2.1. Bacterial Strain and Medium

*P. aeruginosa* SW1 stored in our laboratory was grown in nutrient broth at 37 °C and 200 rpm in an orbital shaker until the optical density at 600 nm reached approximately 2.0. The broth culture was then used as the fermentation inoculum. The medium of fermentation contained 40 g/L soybean oil, 5 g/L NaNO_3_, 1.1 g/L KCl, 1.1 g/L NaCl, 4 g/L KH_2_PO_4_, 4.4 g/L K_2_HPO_4_ and 0.5 g/L MgSO_4_·7H_2_O. The medium was sterilized at 121 °C for 20 min for fermentation. Chemicals were purchased from Sinopharm Chemical Reagent Co., Ltd. (Shanghai, China). The bean oil was purchased from Shandong Luhua Group Industry Co., Ltd. (Laiyang, China). In addition, in this study, general reagents and chemicals were purchased from Sinopharm Chemical Reagent Co., Ltd., and their purity was analytical grade, unless otherwise stated.

### 2.2. Fermentation

The seed cultures were inoculated 1:10 into the fermentation medium. Fermentation was carried out in 250 mL Erlenmeyer flasks at 37 °C with orbital shaking at 200 rpm for 96 h. A 7.5 L fermenter with a working volume of 4 L produced by Shanghai Baoxing Bio-Engineering Co, Ltd. (Shanghai, China), was employed. The yield and biomass of rhamnolipid fermentations carried out at pH 5.5 and pH6.0 were detected respectively. The temperature of fermentation was maintained at 37 °C. The pH was controlled by the addition of 3 M HCl or ammonia. Agitation and aeration flux were 300 rpm and 3 L/min, respectively. 

### 2.3. Rhamnolipid Concentration and Biomass 

The yield of rhamnolipids was quantified by the colorimetric determination of sugar moieties with orcinol [16]. The culture broth of rhamnolipids was initially centrifuged (10,000× *g*, 10 min) to separate the cells from the supernatant, and then 0.5 mL of supernatant was extracted three times with 1 mL of chloroform/ethanol (2:1, *v*/*v*). The organic phase was collected and evaporated to dryness, and then the residue of rhamnolipids was dissolved in deionized water. Then, 2.7 mL of the solution containing 0.19% orcinol (in 53% H_2_SO_4_) was added to 0.3 mL of rhamnolipid solution and heated at 80 °C for 30 min, after which the reaction mixture was cooled in an ice–water bath for 2 min and the absorbance of the reaction mixture was measured at 421 nm. Rhamnolipids were quantified in triplicate, and the concentration of rhamnolipids was calculated by multiplying the amount of rhamnose by 3.4 [17]. The dry weight method was employed to measure biomass. The error bars in the figures are the standard deviations of the triplicate experiments.

### 2.4. Rhamnolipid Purification

The purification of rhamnolipids was performed according to Wang’s method with slight modifications [17]. Briefly, the culture medium was centrifuged at 10,000 rpm for 10 min at 4 °C. The supernatant was acidified to pH 2.0 with 6 M hydrochloric solution and stored overnight at 4 °C to precipitate the supernatant. The supernatant was then centrifuged at 10,000 rpm for 1 h at 4 °C to recover the precipitate. The precipitate was then dissolved in deionized water to prepare the rhamnolipid solution, and two volumes of chloroform:ethanol (2:1, *v*/*v*) were added to the rhamnolipid solution and then shaken at room temperature for 1 h for extraction. The organic phase was evaporated to dryness at 40 °C to remove the solvent. After evaporation of the solvent, the residues of rhamnolipids were obtained.

### 2.5. Foaming Properties

The fermentation broths of rhamnolipids (22 g/L, 30 mL) from 250 mL Erlenmeyer flask were controlled by 3 M HCl and ammonia at pH 4, pH 5, pH 6, pH 7 (neutral pH control) and pH 8 (weak alkaline pH control). The fermentation broths were then stirred with a rotor–stator disperser (AM400W-H, Angni Instruments Ltd., Shanghai, China) at 1000 rpm for 2 min at 37 °C, and the foam was poured into a 100 mL graduated cylinder immediately. Foaming ability (FA) was determined by comparing the volume of foam at 1 min with the initial liquid volume of the sample. The foam volume at 5 min was measured to evaluate the foam accumulation capacity.

FA (%) = V_1_/30 × 100 (1)

where V_1_ is the foam volume at 1 min.

### 2.6. Foaming Microstructure Observation by Optical Microscopy

The microstructures of the foam of rhamnolipid fermentation broth at pH 4–8 were observed with a microscope (CX43, Olympus, Tokyo, Japan) according to the method reported by Xiong [18] with slight modifications. Samples (10 μL) were placed on microscopy glass slides, and then the foams were observed after being stored at 37 °C for 1 and 5 min. The magnification was 10 (objective) × 10 (eyepiece).

### 2.7. Composite Mutagenesis of UV and EMS 

UV and EMS compound mutagenesis were carried out using Siegmund Wagner plates with minor modification [19]. Briefly, 0.2 g cetyltrimethylammonium bromide (CTAB) and 0.005 g methylene blue were added to the nutrient broth agar plates, and the pH was adjusted to 5.5 with 3 M HCl and 1 M NaOH. For UV mutagenesis, the collected cells (3000 rpm, 10 min) were resuspended in nutrient broth at approximately 1 × 10^4^ cells mL^−1^. The cells were then mutated with a 30 W UV lamp (245 nm, Shengxing ZW30S19W) at a distance of 30 cm for 5, 10, 20, 30, 40 and 50 s. The irradiated cell suspension was diluted 100-fold with sterilized saline, and then 0.1 mL of dilution was spread on the Siegmund Wagner plates. The plates were incubated at 37 °C for 5 days. All these manipulations were conducted in dark to avoid possible photoreactivation. Strains with large blue circles were isolated for the further EMS mutagenesis. To perform EMS mutagenesis, collected cells (10,000 rpm for 5 min) were resuspended in sterilized saline at approximately 1 × 10^6^ cells mL^−1^. In a dark room, cells were subjected to 5 mL of 2% (*v*/*v*) EMS for more than 0.5 h, and then EMS was inactivated by adding sterile sodium thiosulfate to a final concentration of 5%. Cells were pelleted at 10,000 rpm and washed three times with sterilized saline, and then they were resuspended in nutrient broth at a rate of approximately 1 × 10^6^ cells mL^−1^. Then, 0.1 mL of cell suspension was spread on the Siegmund Wagner plates and incubated at 37 °C for 5 days. 

### 2.8. LC-MS Analysis

Three rhamnolipid purification samples were applied for congener analysis. Sample A was the rhamnolipids obtained from *P. aeruginosa* SW1 without pH control in Erlenmeyer flask fermentation; Sample B was the rhamnolipids obtained from *P. aeruginosa* SW1 by fermentation using a fermenter at pH 5.5; Sample C was the rhamnolipids obtained from mutant strain E7 by fermentation using a fermenter at pH 5.5. These samples were analyzed using a Waters Xevo G2-XS Q-TOF (quadrupole time-of-flight) Mass Spectrometer (Waters Corporation, Milford, MA, USA) coupled with a Waters ACQUITY UPLC equipped in negative mode (Waters Corporation, Milford, MA, USA). The analysis method was based on Déziel’s method [19] with slight modifications. Briefly, a C18 reverse-phase column with 20 µL of sample was used at a flow rate of 0.5 mL/min. An acetonitrile (purity suitable for LC-MS, Merck Millipore, Burlington, MA, USA)–water gradient was used, starting with 10% acetonitrile for 1 min, increasing to 60% acetonitrile in 10 min and holding for 5 min. Ten percent of the flow was introduced into the mass spectrometer. The capillary voltage was set to 3.8 kV, the cone voltage was set to 35 V and the source temperature was kept at 100 °C. The scan mass range was 50–800 Da.

### 2.9. Bioactivity Analysis of Rhamnolipids

An antiproliferative test against cancer cell lines was performed using MTT assay to investigate the effect of changes in fermentation conditions on the biological activity of rhamnolipids. The two cell lines (A549 and Hela) were grown in DMEM at 37 °C, 5% CO_2_. Cultures of the cancer cell lines were supplemented with 10% fetal bovine serum (FBS) and penicillin and streptomycin at 100 units/mL and 0.1 mg/mL, respectively. Cell lines were pooled and dispensed into a 96-well plate. Rhamnolipids from Sample A, Sample B and Sample C were dissolved in ultrapure water, sonicated and then diluted to 20, 40, 60, 80, 100, 140 and 180 mg/L and added to the cells to a final volume of 100 µL per well. After 48 h of incubation, 5 mg/mL MTT was added to each well and incubated for another 4 h, and then 100 µL DMSO was added and mixed thoroughly. Absorbance was measured at 570 nm using a microplate reader. Positive and negative controls were included in the experiment.

### 2.10. Surface Tension, Viscosity and Zeta Potential

The fermentation broth of rhamnolipids from strain E7 in the fermenter was adjusted to pH 5.0, pH 5.5, pH 6.0 and pH 8.0 (control) with 3 M HCl and ammonia. Surface tension was measured by the Wilhelmy plate method using the tensiometer (SFZL-E, Innuo Pecision Instruments Ltd., Shanghai, China) [20]. The Wilhelmy plate was immersed in 20 mL of aqueous phase at 37 °C, and the viscosity of fermentation broths was measured with a viscometer (NDJ-9S, Pingxuan Scientific Instrument CO., Ltd., Shanghai, China) at 60 rpm. Zeta potential was measured with a Zetasizer Nano ZSP (Malvern Instruments Ltd., Malvern, UK). 

### 2.11. The Average Diameter of Particles and Morphology of Rhamnolipids

The average particle diameters of rhamnolipid solutions at pH 5.0, pH 5.5, pH 6.0 and pH 8.0 were measured by dynamic light scattering (DLS, Zetasizer Nano ZSE, Malvern) at 25 °C. Each DLS measurement was repeated three times. The morphology of rhamnolipid residue from strain E7 in the fermenter was observed by transmission electron microscopy (TEM). The rhamnolipid solution sample and 1% aqueous phosphomolybdic acid solution were adjusted to pH 5.0, pH 5.5, pH 6.0 and pH 8.0 (control) with 3 M HCl and ammonia, respectively. A drop of the test rhamnolipid solution sample was placed onto a copper grid, dried naturally and then stained with 1% phosphomolybdic acid aqueous solution of the same pH for 2 min. The excess phosphomolybdic acid aqueous solution was absorbed with a piece of filter paper, and the grid was then dried naturally. The samples were imaged under a transmission electron microscope (JEM-2100Plus, JEOL, Tokyo, Japan).

### 2.12. Method of Statistical Calculations

The experiments in “2.2. Fermentation”, “2.3. Rhamnolipid Concentration and Biomass”, “2.5. Foaming Properties”, “2.9. Bioactivity Analysis of Rhamnolipids”, “2.10. Surface Tension, Viscosity and Zeta Potential” and the average diameter of particles (“2.11. The Average Diameter of Particles and Morphology of Rhamnolipids”) were repeated three times, and error bars in these studies are the standard deviations of the three repeated experiments. The experiments in “visual foam volume image of rhamnolipid fermentation broths after being stored at 1 and 5 min”, “time evolution of air bubbles formed by rhamnolipid fermentation broths from pH 4–8”, “the TEM micrographs of rhamnolipids at pH 5.0, pH 5.5 and pH 6.0” and “base peak ion intensity chromatograms of rhamnolipids extracted from (A) Sample A and (B) Sample B” were repeated two times, and error bars are the standard deviations of the two repeated experiments; generally representative photos are presented in the corresponding results. 

## 3. Results

### 3.1. Evaluation of Foaming Properties of Rhamnolipid Fermentation Broths at pH 4–8

**Figure 1:**
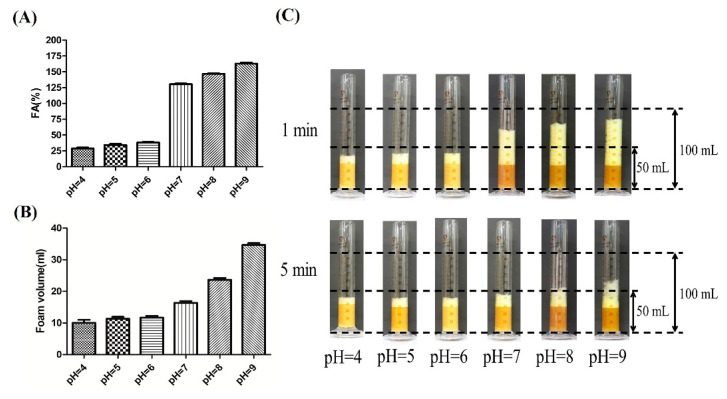
Evaluation of foaming properties of rhamnolipid fermentation broths at pH 4–8. (**A**) The foaming ability evaluation of rhamnolipid fermentation broths; (**B**) the foam volume of fermentation broths of rhamnolipids at 5 min; (**C**) the visual foam volume image of rhamnolipid fermentation broths after being stored at 1 and 5 min. The black dashed lines of the same height in (**C**) represent the same volume of the graduated cylinder.

**Figure 2:**
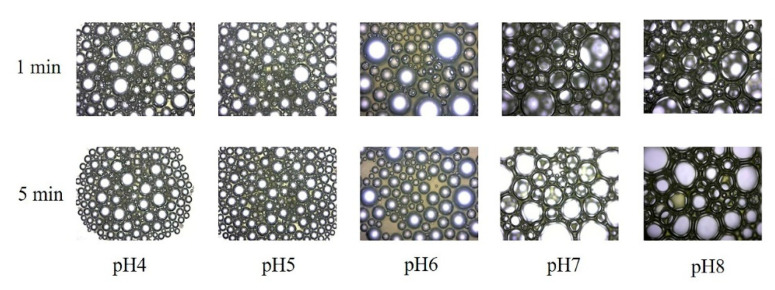
Time evolution of air bubbles formed by rhamnolipid fermentation broths with pH 4–8. All images were obtained at the same magnification.

The fresh fermentation broths without centrifugation were employed to evaluate the fermentation foaming properties of *P. aeruginosa* SW1. As shown in Figure 1A,C, the FAs of fermentation broths at pH 4, pH 5, pH 6, pH 7 and pH 8 were 32%, 41%, 42%, 146% and 174%, respectively. After being stored for 5 min, there was less foam in the pH 4, pH 5 and pH 6 fermentation broths than in the pH 7 and pH 8 broths (Figure 1B), indicating the foaming behavior of rhamnolipid fermentation broths can be significantly suppressed under weakly acidic conditions. In addition, the time evolution of bubbles (Figure 2) shows that the bubbles at pH 4–6 have lesser layers at 1 min, and most of the foaming had ruptured with only a few small-sized bubbles present in the field of view at 5 min. In contrast, when being stored for 5 min, the rhamnolipid fermentation broth with pH 7–8 still had more multilayered bubbles with larger bubble sizes, indicating that the fresh fermentation broth with pH 4–6 is less capable of foaming and preventing drainage or coarsening than that with pH 7–8. 

### 3.2. Evaluation of Foaming Behavior of Rhamnolipid Fermentation at pH 5.5 and pH 6.0

**Figure 3:**
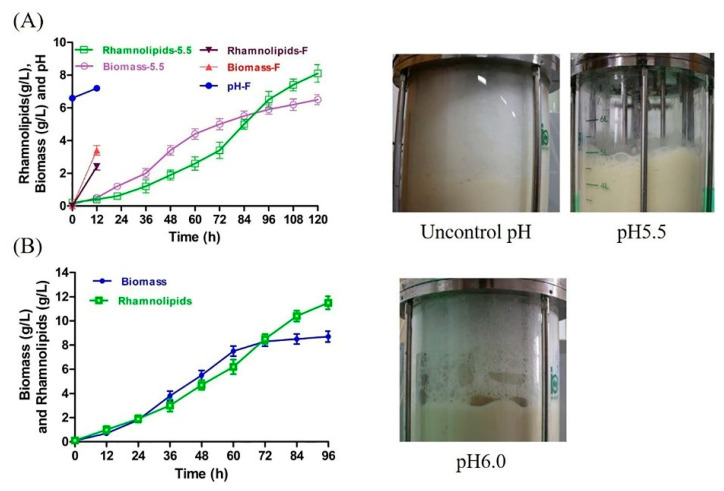
Rhamnolipid yield, biomass and digital images of rhamnolipid fermentation. (**A**) Rhamnolipid yield, biomass and digital images of fermentations carried out at uncontrolled pH and pH 5.5. Biomass-5.5 and rhamnolipids-5.5 represent the biomass and rhamnolipid production at pH 5.5, respectively; Biomass-F, Rhamnolipids-F and pH-F represent the biomass, rhamnolipid production and pH at uncontrolled pH, respectively. When fermentation was carried out in fermenter without pH control, excessive foam overflowed from the effluent gas outlet of the fermenter, forcing the fermentation to stop at 12 h. (**B**) Rhamnolipid yield, biomass and digital images of fermentation at pH 6.0.

To evaluate the foaming behavior of rhamnolipid fermentation under weakly acidic conditions, rhamnolipid fermentation was firstly conducted at pH 5.5. Interestingly, extremely few foams were produced during fermentation, and the fermentation can be conducted for 120 h or longer (Figure 3A). However, although rhamnolipid production and biomass increased through the entire fermentation, the maximum rhamnolipid production (8.1 g/L) and biomass (6.5 g/L) were lower than those in the Erlenmeyer flask fermentation (22 g/L and 12.9 g/L, Figure S1). In addition, after fermentation, some viscous substances similar to mud were found to adhere to the agitator shaft of the fermenter (the picture is not shown) but were not found in the fermentation under uncontrolled pH conditions in our previous study [14]. 

To improve the yield of rhamnolipids, the pH of fermentation was adjusted to 6.0. The yield and biomass of rhamnolipids are plotted in Figure 3B. The maximum rhamnolipid yield (12.5 g/L) and maximum biomass were 0.5-fold and 0.3-fold higher than those of pH 5.5, respectively. However, although the antifoam agent was added at 60, 72 and 86 h, severe foam still occurred during fermentation (Figure 3B), causing foam to overflow from the exhaust pipe. Given that the overflow of cultivation is unacceptable during fermentation [21], in the later experiments, the fermentation of rhamnolipids was carried out at pH 5.5. 

### 3.3. Rebounded Rhamnolipid Productivity after UV and EMS Compound Mutagenesis at pH 5.5

In order to rebound rhamnolipid productivity at pH 5.5, *P. aeruginosa* SW1 was first mutagenized by UV irradiation and then screened with the Siegmund Wagner plates at pH 5.5. On Siegmund Wagner plates, rhamnolipids produced by a single colony can form a blue circle around the colony [19]. The blue circle diameter/colony diameter (BC) is proportional to the rhamnolipid production capacity of strains. To obtain the mutant strains with increased rhamnolipid yield, 20 mutant colonies with large blue circles were screened and the values of BC were calculated. As shown in Table S1, all the mutant strains shown high values of BC compared to the wild-type strain (WT). Strain U3 showed the highest value of BC with a blue circle diameter of 16 mm and a colony diameter of 7 mm. The higher BC values of strain U8 and strain U15 indicate that they also have relatively high productivity. Therefore, strain U3, strain U8 and strain U15 were isolated for EMS mutagenesis. In EMS mutagenesis, strain U3, strain U8 and strain U15 were mutated, and 20 colonies with large values of BC were screened. As shown in Table S1, the highest value of BC was found for strain E7, followed by strain E18, strain E15, strain E19 and strain E8, indicating that the rhamnolipid yields of four strains are significantly improved by mutagenesis. Therefore, we selected the above four strains for further study.

The high yield performance of strains obtained by mutagenesis may be unstable, as a reversal of mutations often occurs during subculture. To assess the stability of production, strain E7, strain E8, strain E15, strain E18 and strain E19 were each cultured in the 7.5 L fermenter with 2 L initial fermentation medium. After that, 1 L of fermentation liquid was removed, and simultaneously, 1 L of fresh fermentation medium was added by timer control digital pump every 48 h for continuous subculture. The pH 5.5 was controlled by the addition of 3 M HCl or ammonia. Agitation was fixed at 200 rpm and aeration flux was set at 2 L/min. The yield of rhamnolipids was detected at 240, 288 and 336 h. As shown in Figure S2, after 336 h of continuous subculture, all strains produced more than 11 g/L of rhamnolipids, and strain E7 still exhibited the highest yields of rhamnolipids (13.6 g/L) followed by strain E15 (12.9 g/L) and strain E18 (12 g/L). 

**Figure 4:**
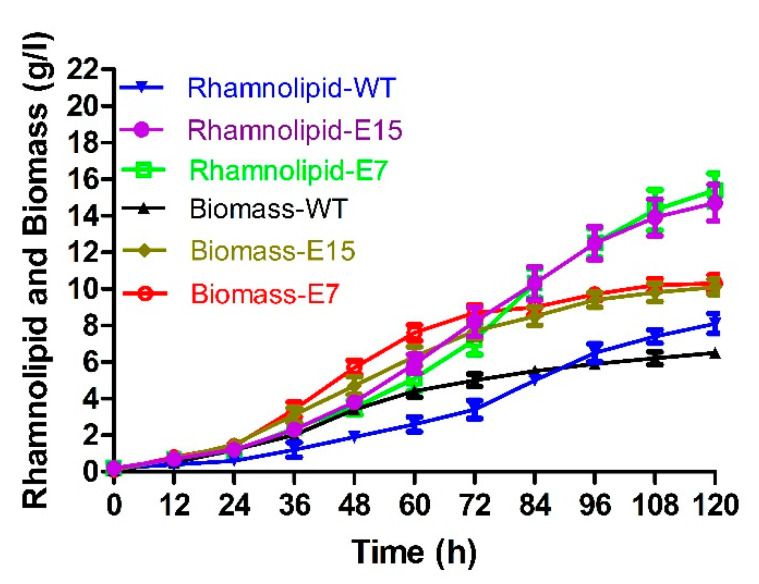
Rhamnolipid yield and biomass of strain E7, strain E15 and wild-type strain at pH 5.5.

Strain E7 and strain E15 were selected for the evaluation of fermentation at pH 5.5. As shown in Figure 4, after 120 h of fermentation, the rhamnolipid production (15.4 g/L) and biomass (10.3 g/L) of strain E7 increased about 0.9-fold and 0.6-fold compared to those of *P. aeruginosa* SW1 at pH 5.5, and the rhamnolipid yield (14.7 g/L) and biomass (10.1 g/L) of the strain E15 increased about 0.8-fold and 0.6-fold compared to those of *P. aeruginosa* SW1 at pH 5.5. Although rhamnolipid production in the fermentation broth was increased continuously, the fermentation still exhibited the “non-foaming” behavior throughout the fermentation process, indicating that fermentation at pH 5.5 is a promising strategy to solve the bottleneck of foaming in rhamnolipid fermentation. 

### 3.4. Characterizing the Effects of Rhamnolipid Congeners on Foaming Behavior

**Figure 5:**
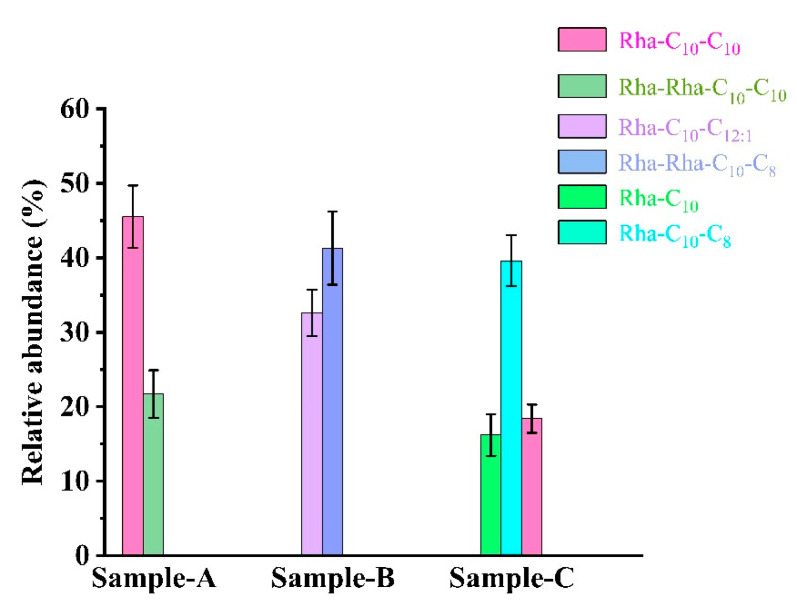
The main structures and relative abundances of rhamnolipids obtained from Sample A, Sample B and Sample C. Sample A: the rhamnolipid sample obtained from *P. aeruginosa* SW1 in Erlenmeyer flasks without pH control. Sample B: the rhamnolipid sample obtained from *P. aeruginosa* SW1 by fermenter with fermentation controlled at pH 5.5. Sample C: the rhamnolipid sample obtained from mutant strain E7 by fermenter with fermentation controlled at pH 5.5.

The congeners of rhamnolipids broths from the strain SW1 and mutant strain E7 were characterized using a Waters Xevo G2-XS Q-TOF Mass Spectrometer coupled with a Waters ACQUITY UPLC equipped in negative mode to assess the effect of congeners on production and foaming behavior. Figure S4 and Figure 5 show the main structures and relative abundance of rhamnolipid congeners obtained from Sample A, Sample B and Sample C. Based on the m/z values of rhamnolipids described in previous studies [22], two rhamnolipid homologs, Rha-C10-C10 and Rha-Rha-C10-C10, are mainly found in Sample A, but Rha-Rha-C_10_-C_8_ (or Rha-Rha-C_8_-C_10_) and Rha-C_12:1_-C_10_ (or Rha-C_10_-C_12:1_) develop into the main rhamnolipid homologs in Sample B. In Sample C, the major species of rhamnolipid homologs increased from two to three, which were Rha-C_10_, Rha-C_10_-C_8_ (or Rha-C_8_-C_10_) and Rha-C_10_-C_10_. 

### 3.5. Characterizing the Bioactivity of the Rhamnolipids from Different Stains and pH Conditions 

**Figure 6:**
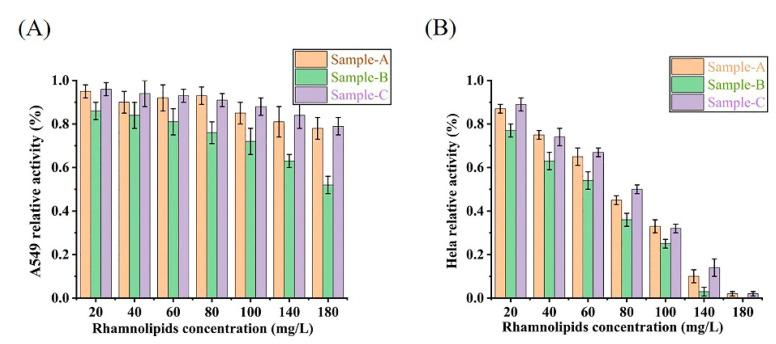
The effects of rhamnolipid Sample A, Sample B and Sample C on cell viability of A549 (**A**) and Hela cells (**B**).

To evaluate the bioactivity of the rhamnolipids from different stains and pH conditions, the effects of rhamnolipid sample A (*P. aeruginosa* SW1, pH 8.2), sample B (*P. aeruginosa* SW1, pH 5.5) and sample C (*P. aeruginosa* E7, pH 5.5) on Hela cell viability and A549 cell viability were tested. As shown in Figure 6, rhamnolipids obtained from Sample A, Sample B and Sample C had significant cytotoxicity against Hela cell lines. The viability of Hela cells treated with increasing rhamnolipid concentrations from all three samples represented a dose-dependent decrease. The IC50 values of Sample A, Sample B and Sample C against Hela cells are 69 mg/L, 61.3 mg/L and 79.8 mg/L, respectively. Rhamnolipids obtained from Sample A and Sample C were not significantly cytotoxic against the A549 cell line when the concentration of rhamnolipids ranged from 20 to 180 mg/L, but rhamnolipids obtained from Sample B had a slight inhibitory effect on the A549 cell line with the IC50 > 180 mg/L.

### 3.6. Characterizing the Effects of pH on Surface Tension and Viscosity 

**Figure 7:**
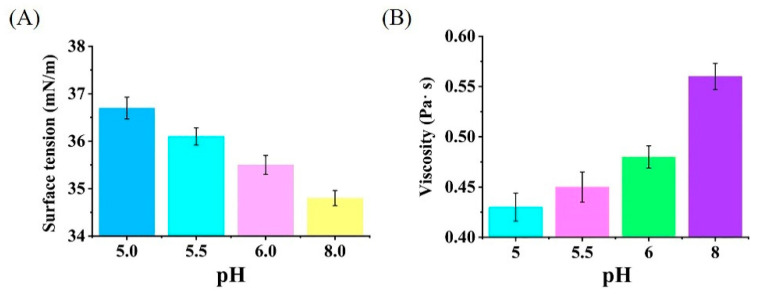
Characterizing the effects of pH on surface tension (**A**) and viscosity (**B**) of rhamnolipid fermentation broths.

As shown in Figure 7A,B, the surface tension increased from 34.8 mN/m to 36.7 mN/m and the viscosity decreased from 0.56 Pa· s to 0.43 Pa· s when the pH of rhamnolipid fermentation broths decreased from 8.0 to 5.0, which shows that the fermentation broths of rhamnolipids under weakly acidic conditions have higher surface tension and lower viscosity than those under weakly alkaline conditions. 

### 3.7. Characterizing the Effects of Zeta Potential and Aggregation Behavior of Rhamnolipids on Foaming Ability 

**Figure 8:**
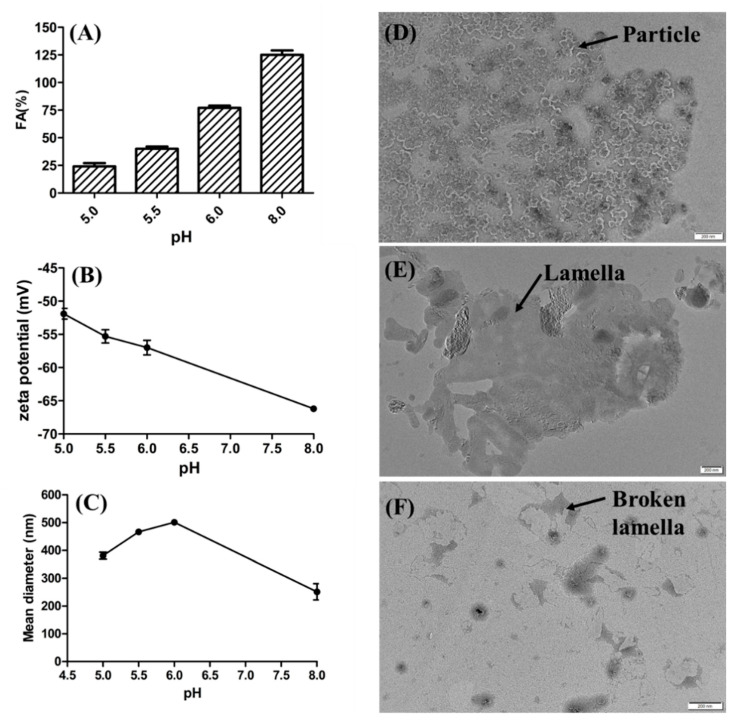
Characterizing the effects of zeta potential and aggregation behavior of rhamnolipids on foaming behavior. (**A**) Foaming ability (FA) of rhamnolipid solutions; (**B**) zeta potential of rhamnolipid solutions; the average diameter of particles of rhamnolipid solutions at pH 5.0−8.0 (**C**); TEM micrographs of rhamnolipids at pH 5.0 (**D**), pH 5.5 (**E**) and pH 6.0 (**F**).

The zeta potential and FA of rhamnolipid solutions were also measured at pH 5.0, pH 5.5, pH 6.0 and pH 8.0 (control). As shown in Figure 8A,B, as the pH value decreased from 8.0 to 5.0, the zeta potential decreased from −66.2 mV to −51.9 mV and the FA of rhamnolipid solutions decreased from 125% to 24%. According to Figure 8A,B, the foaming ability and zeta potential of rhamnolipid solution are simultaneously reduced with the decrease in pH. In addition, there are significant aggregations of rhamnolipid molecules and lower foaming behavior of rhamnolipids at pH 5.0 (Figure S3). The average particle diameters of rhamnolipid solutions at pH 6.0, pH 5.5 and pH 5.0 were 501.0 nm, 466.8 nm and 381.3 nm, respectively (Figure 8C). TEM micrographs of rhamnolipids showed that structures of particulates (vesicles) were presented at pH 5.0 (Figure 8D), and lamellar structures and broken lamellar structures were presented at pH 5.5 (Figure 8E) and pH 6.0 (Figure 8F), respectively. 

## 4. Discussion

According to previous reports, weakly acidic conditions can reduce the foaming behavior of rhamnolipid fermentation, but a systematic evaluation of this is still lacking [15,23]. Furthermore, the biosurfactant rhamnolipids are usually accepted as the major components dominating the severe foaming in the fermentation of rhamnolipids. However, a recent study by Sodagari and Ju claimed that the hydrophobic cells are a more important foaming factor than free rhamnolipids in rhamnolipid fermentation [23]. Therefore, given the effect of cells, secreted proteins and residual soybean oil on the foaming properties of rhamnolipid fermentation, we systematically evaluated the foaming properties of *P. aeruginosa* SW1 by using the fresh fermentation broth without centrifugation at pH 4–8. The results shown in Figure 1 and Figure 2 indicate that the foaming behavior of rhamnolipid fermentation broth is typically pH-dependent and can be significantly suppressed at pH 4–6. As shown in Figure 3A, extremely little foam was produced and the fermentation could proceed for 120 h or longer at pH 5.5, which is significantly longer than the fermentation time without pH control (less than 12 h, Figure 3A), indicating that the long-time “non-foaming” fermentation of rhamnolipids was realized by controlling fermentation at pH 5.5. In a comparison of foaming behavior, cell growth and rhamnolipid production at pH 5.5 and pH 6.0, we found that a 0.5 decrease in pH value can cause a significant decline in foaming ability, and the decrease in pH inhibited the cell growth and biosynthesis of rhamnolipids. The reason for this may be that as the amount of proton hydrogen in fermentation solution is increased, more proton hydrogen can enter into the cell cytoplasm, increasing the intracellular acidity and causing damage to DNA and denaturation of essential enzymes, which impairs cell growth and reduces rhamnolipid synthesis [24], and the further study remains to be elucidated. In addition, according to previous reports, the pH value of rhamnolipid fermentation can be changed from weakly acidic to weakly alkaline during fermentation [3,25] owing to the metabolism of *P. aeruginosa* (in this study, the pH was increased from 6.6 to 8.2 (Figure S1)). According to the foaming properties of fermentation broths presented in this study, the change in pH value from weakly acidic to weakly alkaline remarkably enhanced the foaming ability of rhamnolipid fermentation, which is a deficiency of *P. aeruginosa* for rhamnolipid production. 

The foaming ability of a liquid is related to its surface tension and viscosity [7]. The formation of bubbles is the process of increasing the film surface area, and the increase in film surface area implies that the energy of the bubbles system also is increased correspondingly [26]. Therefore, from a thermodynamic point of view, it is clear that high surface tension is not conducive to the formation of bubbles. This is because the liquid film of bubbles with high surface tension has high surface energy, and more work is needed to produce the same foam. In addition, the lower viscosity and strength of the bubble liquid film formed by the low viscosity solution accelerates the rupture of the bubble liquid film [27]. Therefore, in this study, the fermentation broths of rhamnolipids under weakly acidic conditions showed higher surface tension and lower viscosity than those under weakly alkaline conditions, suggesting that the weakly acidic fermentation is conducive to reducing the foaming behavior of rhamnolipid fermentation broths. In addition, during the fermentation at pH 5.5, some viscous substances, presumed to be a mixture of rhamnolipids, cells, proteins, soybean oil and other metabolites, were found to adhere to the agitator shaft of the fermenter, which may reduce the viscosity and foaming behavior of fermentation broths, and the further study remains to be elucidated.

Compared to *P. aeruginosa* SW1, the rhamnolipid yield (15.4 g/L) and biomass (10.3 g/L) of strain E7 increased approximately 0.9-fold and 0.6-fold at pH 5.5, respectively, which is the first report of improved rhamnolipid production under weakly acidic conditions, indicating that *P. aeruginosa* has the promising potential to further enhance production capacity under weakly acidic conditions. Furthermore, considering that the “non-foaming” fermentation of rhamnolipids can be achieved at pH 5.5, improving the productivity of *P. aeruginosa* at pH 5.5 by carrying out other strain transformation strategies such as metabolic engineering and synthetic biology techniques [28,29] is considered as a promising strategy to solve the foaming bottleneck of rhamnolipid fermentation, and the further study remains to be elucidated.

By characterizing the rhamnolipid homologs, we found that the metabolic flux and the homolog types of Sample A (*P. aeruginosa* SW1, pH 8.2) and Sample B (*P. aeruginosa* SW1, pH 5.5) were significantly different, indicating that the decrease in rhamnolipid yield at pH 5.5 was attributed to the low pH inhibiting metabolic fluxes and changing the types of homologs synthesized by rhamnolipids. In addition, comparing the relative abundance and species of rhamnolipid homologs of Sample B and Sample C (*P. aeruginosa* E7, pH 5.5), we concluded that the increase in rhamnolipid yield of strain E7 is due to changes in metabolic flux and an increase in the types of rhamnolipid homologs. Furthermore, considering that there was no difference in foaming during the fermentation process of Sample B and Sample C, it is speculated that changes in homologous species and metabolic flux did not affect the foaming ability of rhamnolipid fermentation. In previous studies, differences in the components of rhamnolipids were found to affect their bioactivity in terms of antitumor activity. Zhao et al. reported that di-rhamnolipids showed significant antiproliferative activity against MCF-7 and H460 cell lines [30]. Christova et al. found that compared to di-rhamnolipids, mono-rhamnolipids were superior in the inhibition of cell viability of HL-60, BV-173, SKW-3 and JMSU-1 cell lines at lower concentrations [31]. In our study, rhamnolipids obtained from Sample B inhibited A549 cell lines and Hela cell lines and showed better bioactivity against A549 and Hela cell lines compared to those from Sample A and Sample C. This may be caused by the difference in the rhamnolipid congeners in the two samples, with Sample B containing mainly di-rhamnolipids and Sample A containing mono-rhamnolipids. 

According to Figure 8 and Figure S3, the decrease in pH decreased the zeta potential and foaming behavior of rhamnolipid solutions and increased the aggregation behavior of rhamnolipid molecules. In addition, according to previous reports, the pKa of rhamnolipid molecules is about 4.3–5.6, and the aggregation behavior of rhamnolipid molecules can change from lamellar to vesicular at pH 6.0 to pH 5.0 [32], which is practically consistent with our results shown in Figure 8D–F. Therefore, it is considered that the decrease in foaming behavior is partly attributed to the rhamnolipid molecule aggregations caused by the reduced charge potential, and the reason may be as follows: When the pH value is lowered from 6.0 to 5.0, a smaller fractions of rhamnolipids will be present as negatively charged ions because carboxylic groups will be protonated at low pH values [33,34]. The decrease in negatively charged ions can weaken the electrostatic repulsion between rhamnolipids, resulting in aggregations of rhamnolipid molecules from lamellas to particles or vesicles. The aggregations of rhamnolipids cause the uneven distribution of rhamnolipid molecules, reducing the strength of the membranes of bubbles and leading to the rupture of bubbles, which represses the foaming behavior of rhamnolipid solutions [23,35]. On the other hand, the decrease in negatively charged ions also weakens the electrostatic repulsion of charged cells adsorbed on the inner and outer membranes of bubbles, making the liquid membrane prone to draining, thinning and rupture [36].

In the previous findings, there is a paradox that rhamnolipid solutions are characterized by low foaming capacity, but the rhamnolipid fermentation is severely foaming [37,38]. The reason may be explained by this study, where the rhamnolipid solution is weakly acidic (pH about 4.5 in this study) [23,32], resulting in the low foaming of rhamnolipid molecules. However, rhamnolipid fermentation is generally carried out under weakly alkaline conditions caused by the metabolism of *P. aeruginosa* (Figure 3A), enhancing the foaming ability of rhamnolipids. Therefore, the pH of the rhamnolipid solution should be adjusted to neutral or alkaline when the solution is used as a foaming agent, as an emulsifier, as an oil recovery agent or in other areas.

## 5. Conclusions

In this study, the foaming properties of rhamnolipid fermentation broths under weakly acidic conditions were systematically evaluated, and the results indicated that the foaming behavior of rhamnolipid fermentation broths can be significantly inhibited by weakly acidic conditions, and the “non-foaming” fermentation can be achieved by controlling the rhamnolipid fermentation at pH 5.5, but the yield decreased significantly. Further, rhamnolipid production was rebounded 1.9-fold by UV and EMS compound mutagenesis at pH 5.5, indicating *P. aeruginosa* has the promising potentiality to further improve rhamnolipid production by genetic modification under weakly acidic conditions, which was reported for the first time. The homolog types and major rhamnolipid homologs synthesized by *P. aeruginosa* were significantly changed by the UV and EMS compound mutagenesis, but this hardly affected the foaming behavior and bioactivity of rhamnolipids. Mechanistic studies suggest that the reduced foaming ability of rhamnolipids with decreased pH may be attributed to the increase in surface tension and the decline in viscosity and electrostatic repulsion, as well as aggregation behavior change of rhamnolipid molecules. This work proved a possible way for overcoming the bottleneck of foaming in rhamnolipid fermentation and realizing efficient production of rhamnolipids. 

## Data Availability

All datasets generated for this study are included in the article/Supplementary Materials.

## Supplementary Materials

The following supporting information can be downloaded at: https://www.mdpi.com/article/10.3390/microorganisms10061091/s1, Figure S1: Kinetics of the pH, biomass and rhamnolipids of fermentation by *Pseudomonas aeruginosa* SG1 in 250 mL Erlenmeyer flask (EF). Figure S2: The production of rhamnolipids of strains E7, E8, E15, E18 and E19 at pH 5.5. Figure S3: The visual foam volume image of rhamnolipid solution after being stored for 1 min. Figure S4: Base peak ion intensity chromatograms of rhamnolipids extracted from (A) Sample A, (B) Sample B and (C) Sample C. Table S1: Blue circle diameters/colony diameters of strains.
