# Supplementary material for: Achieving “Non-Foaming” Rhamnolipid Production and Productivity Rebounds of Pseudomonas aeruginosa under Weakly Acidic Fermentation
Source: Microorganisms. 2022 May 25;10(6):1091. doi: 10.3390/microorganisms10061091 (PMC9227327; doi:10.3390/microorganisms10061091)
Supplement: Supplementary file 1 [file microorganisms-10-01091-s001.zip › microorganisms-1732362-supplementary.pdf]

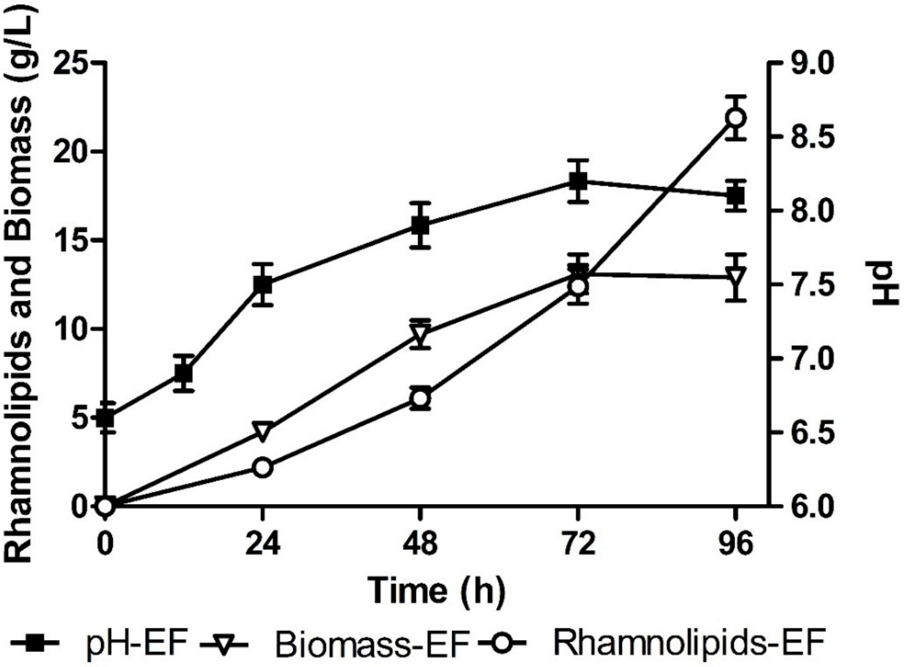

Figure S1. Kinetics of the pH, biomass and rhamnolipids of fermentation by *Pseudomonas aeruginosa* SG1 in 250 mL Erlenmeyer flask (EF).

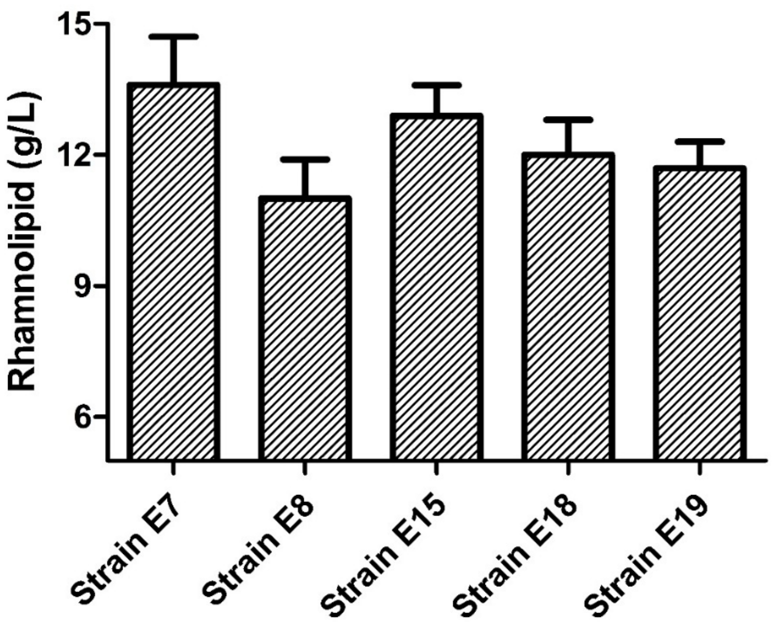

Figure S2. The production of rhamnolipids of strain E7, E8, E15, E18 and E19 at pH 5.5.

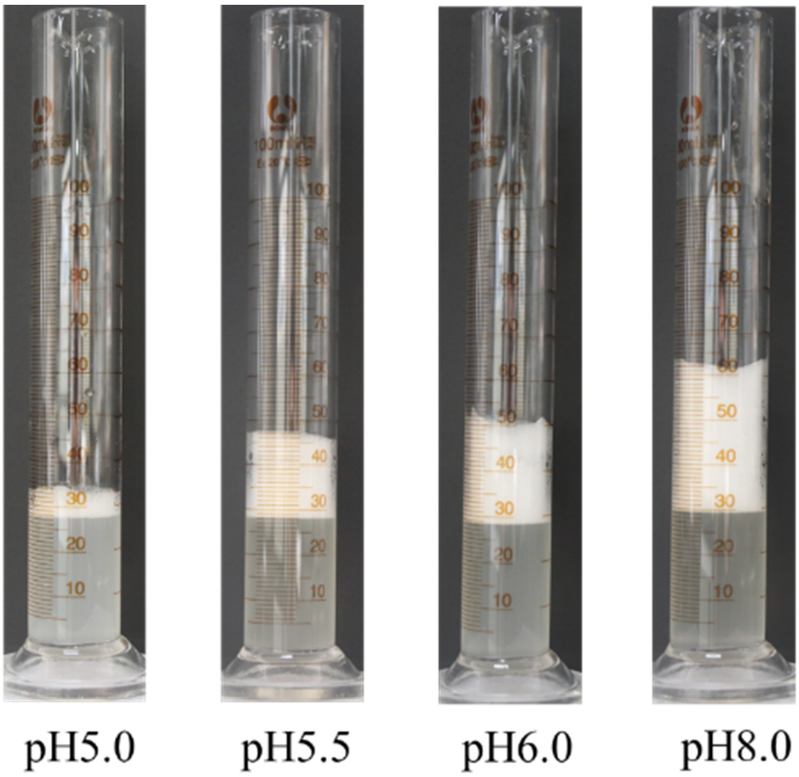

Figure S3. The visual foam volume image of rhamnolipids solution after being stored for 1 min.

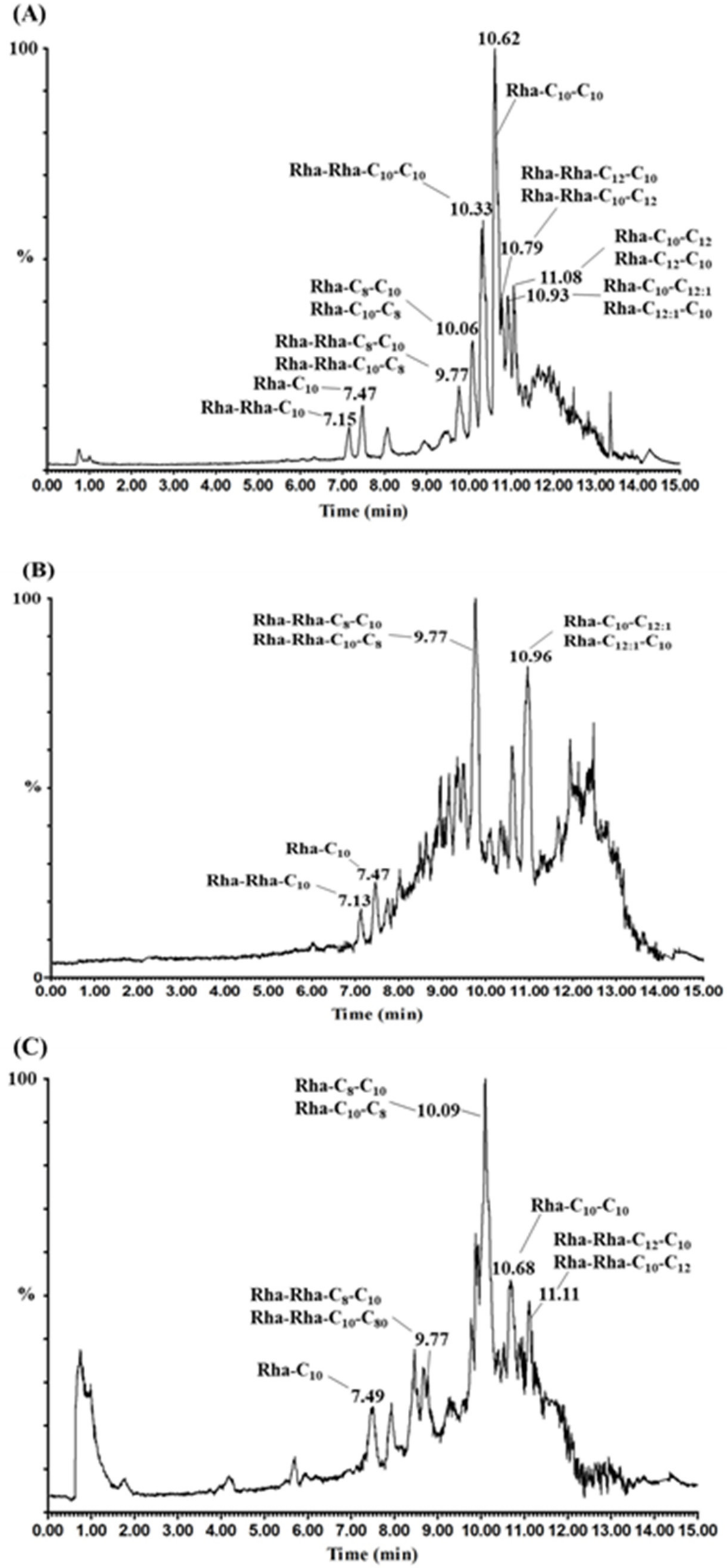

Figure S4. Base peak ion intensity chromatograms of rhamnolipids extracted from (A) Sample-A, (B) Sample-B and (C) Sample-C.

Table S1. Blue circle diameters/Colony diameters of strains

UV				EMS			
Code	R1(mm)	R2 (mm)	BC	Code	R1(mm)	R2 (mm)	BC
WT	6.0	4.0	1.5				
U1	10.0	6.5	1.5	E1	14.5	7.0	2.1
U2	9.5	5.0	1.9	E2	19.0	7.5	2.5
U3	16.0	7.0	2.3	E3	15.0	6.0	2.5
U4	7.5	4.0	1.9	E4	19.0	9.0	2.1
U5	9.0	5.0	1.8	E5	15.5	7.0	2.2
U6	16.5	9.0	1.8	E6	14.0	6.5	2.2
U7	9.5	5.5	1.7	E7	21.0	7.0	3.0
U8	13.0	6.0	2.2	E8	16.0	6.0	2.7
U9	8.5	5.0	1.7	E9	17.0	7.0	2.4
U10	8.0	4.5	1.8	E10	15.0	6.0	2.5
U11	9.0	5.5	1.6	E11	18.0	7.5	2.4
U12	11.5	6.5	1.8	E12	17.0	6.5	2.6
U13	8.0	5.0	1.6	E13	19.0	7.5	2.5
U14	12.5	6.0	2.1	E14	18.0	8.0	2.3
U15	14.5	6.5	2.2	E15	18	6.5	2.8
U16	14.5	8.5	1.7	E16	19.0	7.5	2.5
U17	9.5	5.0	1.9	E17	17.0	6.5	2.6
U18	13.0	7.5	1.7	E18	20.0	7.0	2.9
U19	9.0	5.0	1.8	E19	17.0	6.0	2.8
U20	13.0	7.5	1.7	E20	18.0	7.0	2.6

R1: Blue circle diameters; R2: Colony diameters; BC: R1/ R2. WT is the wild-type strain (without mutation).
